# AMPK-Mediated Hypolipidemic Effects of a *Salvia miltiorrhiza* and *Paeonia lactiflora* Mixed Extract on High-Fat Diet-Induced Liver Triglyceride Accumulation: An In Vivo and In Vitro Study

**DOI:** 10.3390/nu16183189

**Published:** 2024-09-20

**Authors:** Juah Son, Nguyen Viet Phong, Mi-Ran Cha, Byulnim Oh, Sukjin Song, Seo Young Yang

**Affiliations:** 1 USCAREPHARM Co., Ltd., Suwon 16690, Republic of Korea; juah@uscarepharm.com (J.S.); mrcha@uscarepharm.com (M.-R.C.); aster@uscarepharm.com (B.O.); 2 Department of Biology Education, Teachers College and Institute for Phylogenomics and Evolution, Kyungpook National University, Daegu 41566, Republic of Korea; ngvietphong@gmail.com

**Keywords:** *Salvia miltiorrhiza*, *Paeonia lactiflora*, triglyceride accumulation, hypolipidemic effects, high-fat diet, hepatic steatosis, AMPK

## Abstract

Background: This study investigates the hypolipidemic effects of a mixed extract of *Salvia miltiorrhiza* and *Paeonia lactiflora* (USCP119) in HFD-fed hamsters and in vitro cellular models. Methods: Over an 8-week period, HFD-fed hamsters were assigned to one of six groups: normal diet, HFD control, HFD with 50 mg/kg USCP119, HFD with 100 mg/kg USCP119, HFD with 50 mg/kg USCP119 twice daily (BID), and HFD with omega-3 fatty acids. Key outcomes assessed included body weight, serum triglycerides (TG), total cholesterol (TC), liver weight, hepatic TG levels, and epididymal fat. In cellular models, the impact of USCP119 on lipid accumulation and adipogenic markers was evaluated. Results: USCP119 treatment at 50 mg/kg BID resulted in the lowest weight gain (15.5%) and the most significant reductions in serum TG and hepatic TG levels compared to the HFD control. The 100 mg/kg dose also led to substantial reductions in serum TG and TC levels and notable decreases in low-density lipoprotein cholesterol. USCP119 at 50 mg/kg once daily reduced TG and TC levels but was less effective than the higher doses. In cellular models, USCP119 was non-toxic up to 400 µg/mL and effectively reduced lipid accumulation, modulated adipogenic markers, and enhanced AMPK signaling, improving lipid metabolism and insulin sensitivity. Conclusions: All USCP119 treatments demonstrated effectiveness in managing hyperlipidemia and related metabolic disorders, with variations in impact depending on the dosage. The ability of USCP119 to reduce fat accumulation, improve lipid profiles, and enhance insulin sensitivity highlights its potential as a valuable dietary supplement for addressing high-fat diet-induced hyperlipidemia and metabolic disturbances.

## 1. Introduction

Non-alcoholic fatty liver disease (NAFLD) encompasses a range of liver conditions characterized by the excessive accumulation of lipids in hepatocytes [1,2]. Affecting nearly 30% of adults, NAFLD has become a significant public health concern, particularly in the United States, where its prevalence continues to rise [3]. The initial stage of NAFLD is marked by triglyceride accumulation in the liver, which is closely linked with hyperlipidemia [4]. As the disease progresses, hepatic steatosis can advance to more severe conditions, such as steatohepatitis, cirrhosis, and ultimately hepatocellular carcinoma [5]. While current treatments primarily emphasize weight reduction to counteract hepatic steatosis, identifying alternative strategies is imperative due to the insufficient understanding of the mechanisms driving lipid accumulation in the liver.

Recent research identifies the consumption of sugar-sweetened beverages and processed foods as a significant risk factor for NAFLD [6,7]. These products, rich in various forms of fat, contribute to increased hepatic triglyceride levels and systemic hypertriglyceridemia. Such metabolic disturbances are associated with impaired glucose and lipid metabolism and heightened inflammation [8,9]. The accumulation of hepatic lipids and the subsequent development of hypertriglyceridemia, which affects 15–20% of the adult population, are linked to an elevated risk of cardiovascular disease [10]. At the molecular level, high fat intake stimulates key transcription factors such as ChREBP and SREBP-1, which orchestrate lipid synthesis by regulating target enzymes like ACC and FAS [11]. These transcription factors play crucial roles in the pathogenesis of NAFLD by modulating lipid homeostasis, highlighting the complex interplay between dietary habits and liver health.

In this context, adenosine monophosphate-activated protein kinase (AMPK) emerges as a key regulator of energy metabolism, influencing both glucose levels and lipid uptake [12]. AMPK is found in multiple tissues such as liver, muscle, heart, and brain cells. It exerts its effects by phosphorylating and thereby deactivating enzymes responsible for the synthesis of cholesterol and fatty acids [13]. Moreover, AMPK acts as an upstream regulator of PPARγ and CEBPα, inhibiting the differentiation of pre-adipocytes into adipocytes [14]. Therefore, the activation of AMPK leads to a reduction in cellular cholesterol, fatty acids, and key molecules like SREBP-1 and HMGCR, further linking its role in managing lipid metabolism with the broader mechanisms underlying NAFLD [15].

Given the complex interplay between lipid metabolism, AMPK regulation, and the progression of NAFLD, there is an urgent demand for innovative treatment approaches. Therefore, this study explores the anti-hyperlipidemic effects and mechanisms of a combined extract of *Salvia miltiorrhiza* and *Paeonia lactiflora*, both in vitro and in vivo. Traditionally used in East Asia, these plants are known for their diverse health benefits [16,17]. *S. miltiorrhiza* contains various bioactive compounds, such as salvianolic acid and tanshinone, which exhibit antioxidant, anti-inflammatory, and cardioprotective effects [18,19]. Similarly, *P. lactiflora* is rich in diverse metabolites, such as paeoniflorin and paeonol, which display anti-inflammatory, antidiabetic, and neuroprotective activities [20,21]. This study specifically examines the effects of a mixed extract of these two medicinal plants on high-fat diet (HFD)-induced NAFLD and hyperlipidemia in hamsters, aiming to provide alternative strategies for their treatment.

## 2. Materials and Methods

### 2.1. Plant Material

Roots of *S. miltiorrhiza* Bunge and *P. lactiflora* Pall. in dried form were procured from Saemmulnaegi Farm and Gyeongbuk Herbal Medicine Agricultural Cooperative (Yeongcheon, Gyeongsangbuk-do, Republic of Korea) in November 2022. The materials were authenticated by one of the authors (S.Y.Y.), with voucher specimens archived at the Institute for Phylogenomics and Evolution (Codes: KNU-USCP-DS2211 and KNU-USCP-JY2211).

### 2.2. Preparation of the Mixed Extract

The mixed extract was obtained by refluxing a 500.0 g mixture of *S. miltiorrhiza* and *P. lactiflora* in 70% EtOH at 70 °C (ratio 1:1). Subsequently, the extract was concentrated under reduced pressure using an EYELA N-1100 vacuum rotary evaporator (EYELA, Tokyo, Japan), generating 107.5 g of the mixed extract (USCP119) with a yield of 21.5% (*w*/*w*).

### 2.3. Animal Care and Experimental Protocol

Forty 7-week-old male golden Syrian hamsters (*Mesocricetus auratus*) were sourced from Janvier-Labs (Le Genest-Saint-Isle, France). They were housed in pathogen-free cages with controlled conditions: 12 h dark–light cycles, 21 ± 2 °C, and 45–65% humidity. The hamsters had continuous access to standard rodent food and water. After acclimating for 1 week, the animals were allocated to six groups (Gs) (*n* = 7 per group) as follows: G1 was given a normal diet (ND) for 8 weeks; G2 was given a HFD for 8 weeks; G3 and G4 were maintained on an HFD supplemented with USCP119 at 50 and 100 mg/kg BW/day, respectively; G5 was maintained on an HFD supplemented with USCP119 at 50 mg/kg BW/day, administered twice daily (BID) at an interval of 12 h; and G6 was maintained on an HFD supplemented with omega-3 (OM3) fatty acids at 50 mg/kg BW/day. The ND was a purified version of the AIN-93G diet [22], and the HFD was ND supplemented with 10% lard and 0.3% cholesterol. USCP119 was dissolved in water and administered daily by oral gavage. Food consumption was monitored daily, while weight gain was assessed weekly. Following 8 weeks of treatment, blood samples were obtained and analyzed for serum triglycerides (TG) using a TG quantification kit (MAK266, Sigma-Aldrich, St. Louis, MO, USA), total cholesterol (TC) using a TC colorimetric assay kit (EEA026, Invitrogen, Carlsbad, CA, USA), and high- and low-density lipoprotein cholesterol (HDL-C and LDL-C) using HDL-C (EEA012, Invitrogen) and LDL-C (EEA014, Invitrogen) colorimetric assay kits, following the manufacturer’s instructions. Additionally, all hamsters were sacrificed, and tissues were secured for further experiments. Specifically, liver tissue was extracted and weighed; half of it was stained with H&E and Oil red O, while the rest was analyzed for hepatic TG levels. Fat was extracted from the epididymis and weighed, and the diameter of the epididymal white adipocyte (eWAT) was analyzed through H&E staining. Three random images of the H&E-stained sections of epididymal fat tissue were captured, using a microscope at 100× magnification. Subsequently, the diameter of fat cells was measured and averaged, assuming that the fat cells were spherical, as follows:

(1) Average area per cell=S/n

(2) $$Average\;radius\;of\;fat\;cells=\sqrt{\frac{Average\;area\;per\;cell}{\pi }}$$

(3) $$Average\;diameter\;of\;fat\;cells=2\times Average\;radius\;of\;fat\;cells=2\times \sqrt{\frac{S/n}{\pi }}$$

where, *S* = total area of fat cells; *n* = number of fat cells.

### 2.4. Measurement of Liver TG Levels

Liver TG levels were quantified using a TG assay kit (MAK266, Sigma-Aldrich), following the manufacturer’s guidelines. Detailed procedures are provided in the Supplemental Material.

### 2.5. Cell Culture

3T3-L1 cells (murine preadipocytes) and HepG2 cells (human hepatoma) were sourced from the American Type Culture Collection (Manassas, VA, USA) and cultured under standard conditions. Detailed methodologies for cell culture, differentiation of 3T3-L1 cells, induction of steatosis in HepG2 cells, and lipid analysis are provided in the Supplementary Material.

### 2.6. Cell Viability and Proliferation Assay

Cell viability and proliferation were assessed using the water-soluble tetrazolium (WST)-8 assay (Biomax, Nowon, Seoul, Republic of Korea). Detailed procedures, including cell seeding, USCP119 treatment conditions, and absorbance measurement, are provided in the Supplemental Material.

### 2.7. Western Blot Analysis

Protein extraction, gel electrophoresis, membrane transfer, and antibody incubations were performed as described in the Supplemental Material. Images were captured using an ImageQuant LAS 4000 system from GE Healthcare (Chicago, IL, USA). Band intensities were quantified with ImageJ software version 1.54 (National Institutes of Health, Bethesda, MD, USA) following established procedures [23,24].

### 2.8. Inhibition of AMPK Phosphorylation

After starvation, HepG2 cells were treated with compound C or DMSO, followed by USCP119 or DMSO. Western blot analysis was conducted as detailed in the Supplemental Material.

### 2.9. Statistical Analysis

Data are presented as the mean ± SD. Biochemical assays were performed in at least three independent experiments for each sample. Statistical significance was determined using two-way ANOVA followed by Tukey’s post-hoc test for pairwise comparisons, with a 95% confidence interval (*p* ≤ 0.05) to control for family-wise error rate. The normality of the data was assessed using the Shapiro–Wilk test, and all data met the assumptions of normality required for ANOVA. Two-tailed *t*-tests were also conducted where appropriate. All analyses were performed using IBM SPSS Statistics 27.0.

## 3. Results

### 3.1. Effects of an HFD Administered for 8 Weeks on BW

**Figure 1:**
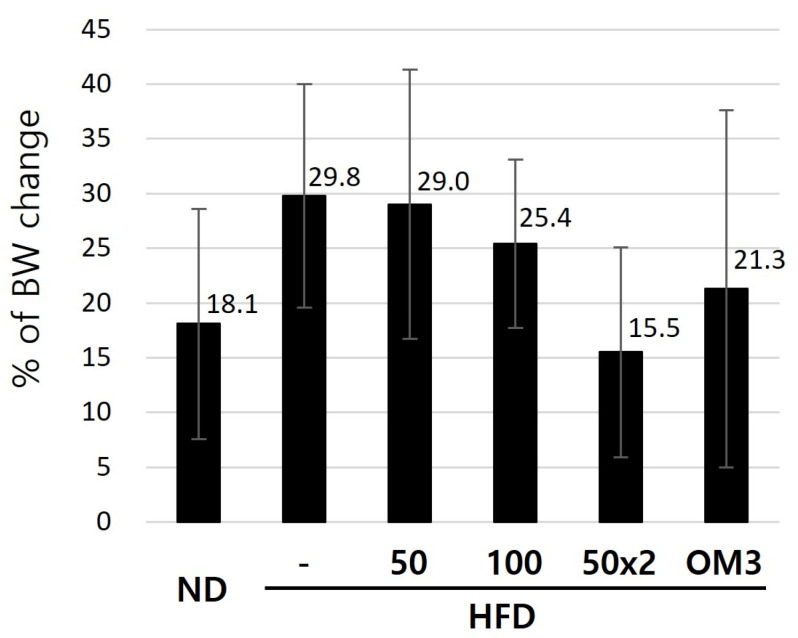
The percentage of body weight changes in hamsters fed a normal diet (ND) or high-fat diet (HFD) with or without USCP119 or OM3 supplementation over 8 weeks.

To investigate the effects of HFD on BW and the potential of oral USCP119 administration to mitigate these effects, hamsters were fed either an ND or an HFD for 8 weeks, with or without daily oral administration of USCP119. OM3 (50 mg/kg/day) served as a positive control. The changes in the BWs of all experimental groups are presented in Figure 1 and Table 1. The initial BWs across all groups were relatively similar, ranging from 103.7 g to 108.8 g, indicating a comparable starting point for the experiment. Over the 8-week period, the ND group (G1) showed an increase in BW from 108.8 g to 126.9 g (18.1% increase), indicating normal growth. The HFD group (G2) experienced a more pronounced increase in BW, from 108.4 g to 138.2 g (29.8% increase), reflecting the impact of an HFD. Group G3, which received an HFD with 50 mg/kg USCP119, exhibited a BW increase comparable to G2, from 105.1 g to 131.1 g (29.0% increase). However, group G4, which received an HFD with a higher USCP119 dose of 100 mg/kg, exhibited a slightly lower increase in BW, from 106.7 g to 132.0 g (25.4% increase), suggesting that a higher dose of USCP119 may mitigate weight gain. Notably, group G5, which received USCP119 at 50 mg/kg BID, exhibited the least weight gain, from 103.7 g to 119.2 g (15.5%). This weight gain was lower than that observed in the HFD group with OM3 supplementation (G6), which had a 21.3% increase. These results demonstrate that while an HFD induces weight gain, supplementation with USCP119 significantly reduces this effect.

### 3.2. Effects of USCP119 on Serum Lipid Profiles in the HFD Hamster Model

**Figure 2:**
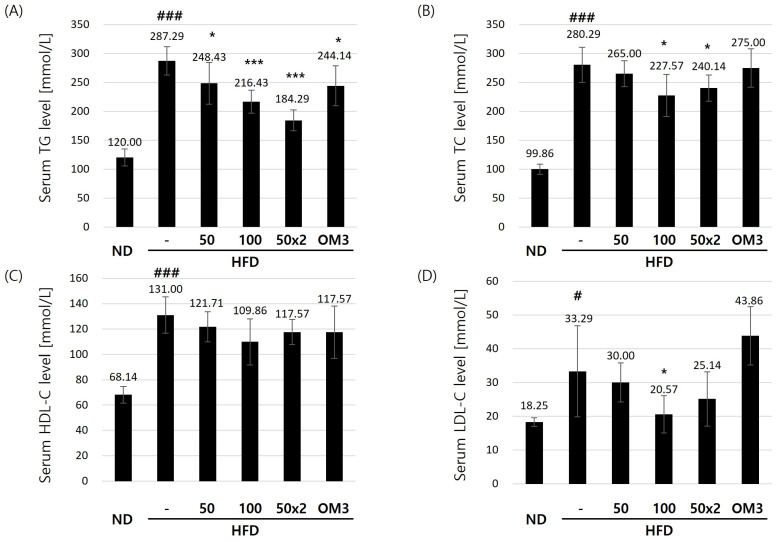
Effects of USCP119 on serum lipid profiles in the HFD hamster model. The levels of (**A**) serum triglycerides (TG), (**B**) serum total cholesterol (TC), (**C**) serum high-density lipoprotein cholesterol (HDL-C), and (**D**) serum low-density lipoprotein cholesterol (LDL-C) in the six experimental groups (*n* = 7; ND–G1, HFD–G2, HFD + USCP119_50–G3, HFD + USCP119_100–G4, HFD + USCP119_50 × 2–G5, and HFD + OM3–G6) after 8 weeks of oral administration. Data are expressed as mean ± SD. ^#^ *p* < 0.05 and ^###^ *p* < 0.001 compared with the ND group (G1); * *p* < 0.05 and *** *p* < 0.001 compared with the HFD group (G2).

Over the 8-week period, the TG levels (Figure 2A and Table 2) in G2, which received HFD alone, were the highest at 287.29 ± 24.64 mmol/L, representing a 239.4% increase compared to the ND group G1. In comparison, group G3, treated with 50 mg/kg USCP119, showed a 13.5% reduction in TG levels (248.43 ± 36.02 mmol/L) compared to group G2. Notably, groups G4 and G5, which received higher doses of USCP119 (100 mg/kg and 50 mg/kg BID, respectively), displayed more significant reductions (*p* < 0.001) in TG levels compared to group G2, with values of 216.43 ± 19.90 mmol/L (24.7% lower than group G2) and 184.29 ± 17.98 mmol/L (35.9% lower than group G2), respectively.

Furthermore, over the 8-week period, the TC levels in group G2 increased significantly (*p* < 0.001) by 280.7% (280.29 ± 30.46 mmol/L) compared to group G1 (99.86 ± 8.88 mmol/L; Figure 2B). Among the USCP119-treated groups, serum TC levels in groups G4 and G5 decreased by 18.8% (227.57 ± 36.41 mmol/L) and 14.3% (240.14 ± 22.59 mmol/L), respectively, compared to group G2 (*p* < 0.05). These results demonstrate that USCP119 substantially reduces serum TC levels.

HDL-C plays a crucial role in lowering blood lipid levels [25]. At the end of the experimental period, group G2, which was fed an HFD, exhibited a 192.3% (131.00 ± 14.40 mmol/L) increase in HDL-C levels compared to the ND group G1 (68.14 ± 6.69 mmol/L). However, no significant differences in HDL-C levels were observed between the USCP119-treated and OM3-treated groups compared to the HFD group G2 (Figure 2C). In contrast, LDL-C levels in all USCP119-treated groups were significantly different from those in the HFD group G2. Specifically, both groups G4 and G5 showed significantly (*p* < 0.05) reduced LDL-C levels: 20.57 ± 5.50 mmol/L (38.2% lower than group G2) and 25.14 ± 8.03 mmol/L (24.5% lower than group G2), respectively (Figure 2D). Notably, OM3 supplementation resulted in the highest LDL-C levels among the HFD-fed groups.

### 3.3. Effects of USCP119 on Hepatic Lipid and Epididymal Fat Levels in the HFD Hamster Model

**Figure 3:**
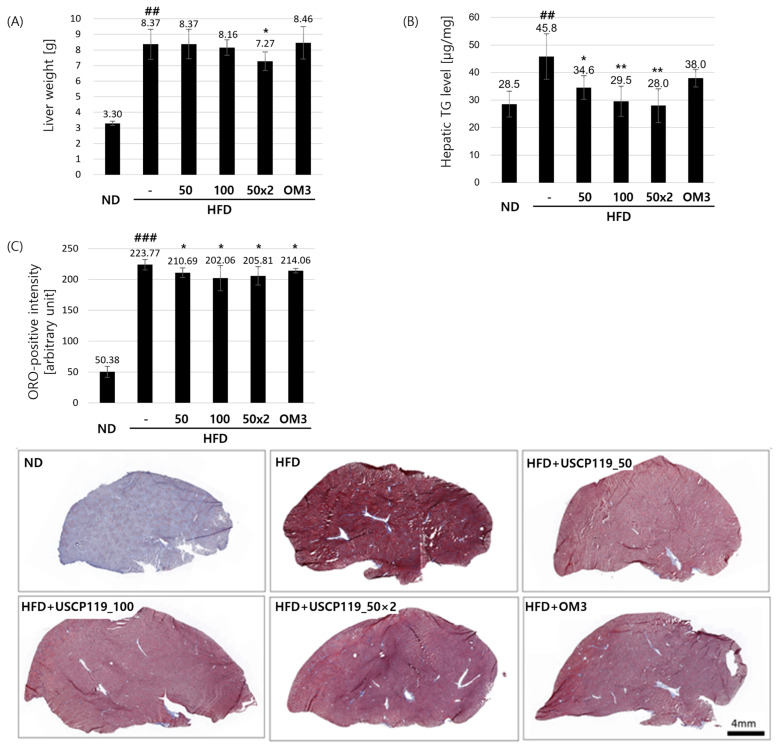
Effects of USCP119 on hepatic lipid levels in the HFD hamster model. (**A**) Liver weights, (**B**) hepatic TG levels, and (**C**) Oil Red O-positive signal quantification results and representative photomicrographs (*n* = 7; ND–G1, HFD–G2, HFD + USCP119_50–G3, HFD + USCP119_100–G4, HFD + USCP119_50 × 2–G5, and HFD + OM3–G6) after 8 weeks of oral administration. Data are expressed as mean ± SD. ^##^ *p* < 0.01, and ^###^ *p* < 0.001 compared with the ND group (G1); * *p* < 0.05 and ** *p* < 0.01 compared with the HFD group (G2).

The impact of USCP119 on the liver and epididymal fat was assessed across the six experimental groups (Figure 3A and Table 3). Liver weight did not differ significantly between the HFD group G2, USCP119-treated groups G3 and G4, and the OM3-treated group G6. However, group G5 exhibited a significant reduction in liver weight (7.27 ± 0.60 g), which was 13.1% lower than that of group G2 (*p* < 0.05). Furthermore, hepatic TG levels were significantly reduced in the USCP119-treated groups compared to the G2 group (Figure 3B). Specifically, groups G4 and G5 had hepatic TG levels of 29.55 ± 5.49 μg/mg (35.6% lower than group G2) and 28.01 ± 6.11 μg/mg (38.9% lower than group G2), respectively.

Oil Red O staining of hamster liver tissues revealed significant lipid accumulation in the livers of the HFD group (G2) compared to those of the ND group (G1) (Figure 3C). The USCP119-treated groups (G3, G4, and G5) and the OM3-treated group (G6) showed notable protection against lipid accumulation, as evidenced by the notable changes in the lipid status of these groups relative to group G2.

**Figure 4:**
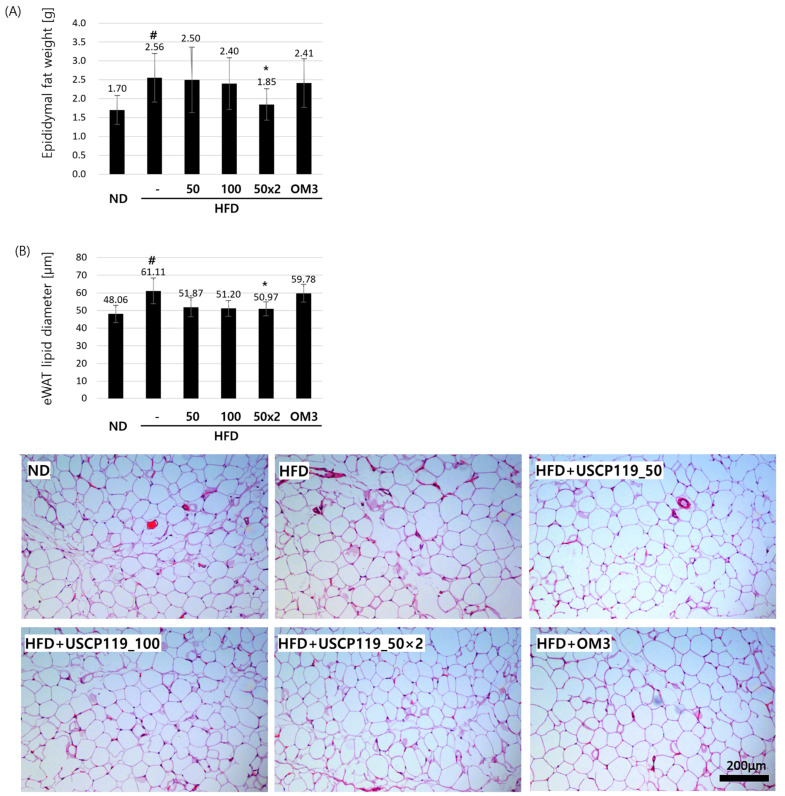
Effects of USCP119 on epididymal fat levels in the HFD hamster model. (**A**) Epididymal fat weights and (**B**) epididymal white adipocyte lipid diameters observed across the six experimental groups (*n* = 7; ND–G1, HFD–G2, HFD + USCP119_50–G3, HFD + USCP119_100–G4, HFD + USCP119_50 × 2–G5, and HFD + OM3–G6) after 8 weeks of oral administration. Data are expressed as mean ± SD. ^#^ *p* < 0.05 compared with the ND group (G1); * *p* < 0.05 compared with the HFD group (G2).

Additionally, HFD significantly increased both epididymal fat weight and the lipid diameter of eWAT (Table 3). The USCP119-treated groups (G3, G4, and G5) demonstrated reductions in fat mass and eWAT lipid diameter compared to the HFD group (G2) (Figure 4A). Notably, group G5, which received 50 mg/kg BID of USCP119, significantly normalized the histopathology of both liver and eWAT tissues, making them resemble those of the ND group (G1). Hypertrophy was prominently observed in the HFD group compared to the ND and USCP119-treated groups (Figure 4B).

### 3.4. Effects of USCP119 on 3T3-L1 Preadipocyte Viability, Lipid Accumulation in Differentiated Cells, and AMPK Signaling Pathways

**Figure 5:**
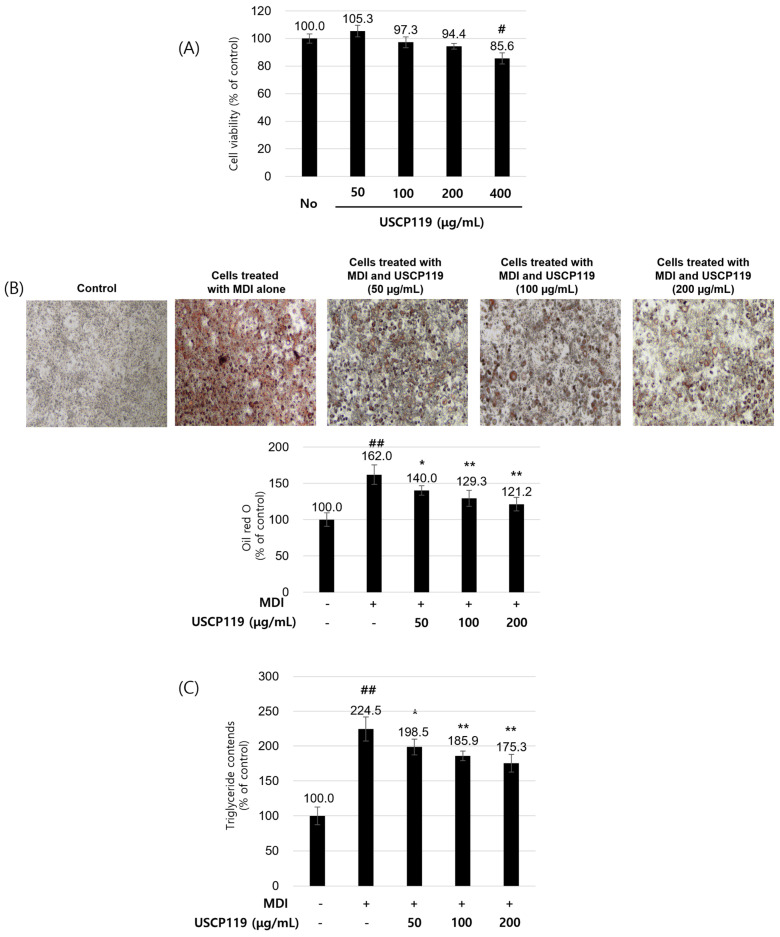
(**A**) Cellular toxicity of USCP119 during the differentiation of 3T3-L1 pre-adipocytes. Cell viability of 3T3-L1 pre-adipocytes was assessed using the MTT assay after 72 h of treatment with or without the indicated doses of USCP119. (**B**) Effects of USCP119 on lipid accumulation during 3T3-L1 pre-adipocyte differentiation. The panel shows representative phase-contrast photographs of Oil Red O-stained cells under 100× magnification. (**C**) Effects of USCP119 on triglyceride deposition in differentiated 3T3-L1 cells. Data are expressed as mean ± SD of three independent experiments (*n* = 3). ^#^ *p* < 0.01 and ^##^ *p* < 0.001 compared with the untreated group; * *p* < 0.05 and ** *p* < 0.01 compared with the MDI (methylisobutylxanthine, dexamethasone, insulin) group.

The impact of USCP119 on 3T3-L1 preadipocytes and differentiated adipocytes was comprehensively investigated, focusing on cell viability, lipid accumulation, TG content, and AMPK signaling pathways. Figure 5A shows that USCP119 maintains high cell viability at concentrations up to 400 µg/mL, indicating that it is non-toxic to 3T3-L1 pre-adipocytes at these levels. Figure 5B demonstrates that USCP119 significantly reduces lipid accumulation in differentiated 3T3-L1 cells, as evidenced by decreased Oil Red O staining in a dose-dependent manner. Figure 5C supports this finding, showing a significant reduction in TG content with increasing USCP119 concentrations.

**Figure 6:**
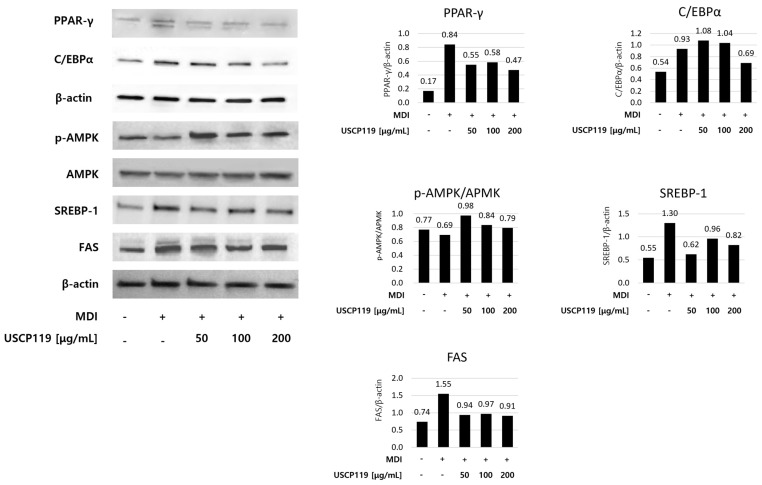
Effects of USCP119 on adipogenic protein expression in 3T3-L1 cells. Data are expressed as mean ± SD of three independent experiments (*n* = 3).

Furthermore, to determine the impact of USCP119 on proteins involved in lipogenesis and lipid degradation, the expression levels of *p*-AMPK, AMPK, PPAR-γ, C/EBPα, SREBP-1, and FAS were assessed using Western blot analysis (Figure 6). During the differentiation of adipose progenitor cells into adipocytes, USCP119 treatment (50, 100, and 200 µg/mL) resulted in a concentration-dependent decrease in the expression of PPAR-γ, C/EBPα, SREBP-1, and FAS, which are typically upregulated during the differentiation process. Additionally, USCP119 treatment increased the phosphorylation of AMPK, which usually decreases during differentiation. These findings indicate that USCP119 inhibits adipogenesis and lipid synthesis through multiple mechanisms, emphasizing its potential as an anti-hyperlipidemic agent.

### 3.5. Effects of USCP119 on HepG2 Cell Viability, Lipid Accumulation in Differentiated Cells, and AMPK Signaling Pathways

**Figure 7:**
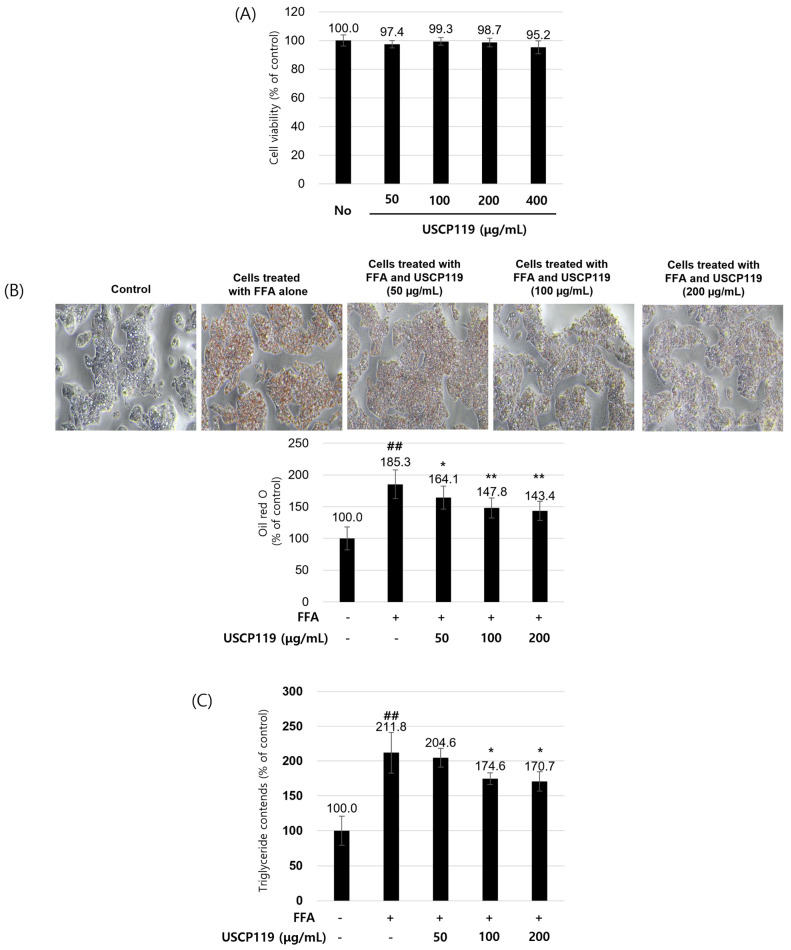
(**A**) Cellular toxicity of USCP119 during the differentiation of HepG2 cells. The cell viability of HepG2 cells was assessed using the MTT assay after 72 h of treatment with or without the indicated doses of USCP119. (**B**) Effects of USCP119 on lipid accumulation in HepG2 cells during differentiation. The panel shows representative phase-contrast photographs of Oil Red O-stained cells at 100× magnification. (**C**) Effects of USCP119 on triglyceride deposition in differentiated HepG2 cells. Data are expressed as mean ± SD of three independent experiments (*n* = 3). ^##^ *p* < 0.001 compared with the untreated group; * *p* < 0.05 and ** *p* < 0.01 compared with the FFA (free fatty acid) group.

The impact of USCP119 on HepG2 cells was also assessed, focusing on cell viability, lipid accumulation, TG content, and AMPK signaling pathways. Figure 7A shows that USCP119 maintains high cell viability at concentrations of 50, 100, 200, and 400 µg/mL, with cell viability percentages consistently close to 100%, indicating its non-toxic nature up to 400 µg/mL. Figure 7B reveals that USCP119 significantly reduces lipid accumulation in HepG2 cells treated with FFAs, as evidenced by the dose-dependent decrease in Oil Red O staining. Figure 7C supports these findings, showing a significant reduction in TG content with increasing USCP119 concentrations. The TG content drops from 211.8% in FFA-treated cells to 174.6% and 170.7% for 100 and 200 µg/mL of USCP119 treatment, respectively.

**Figure 8:**
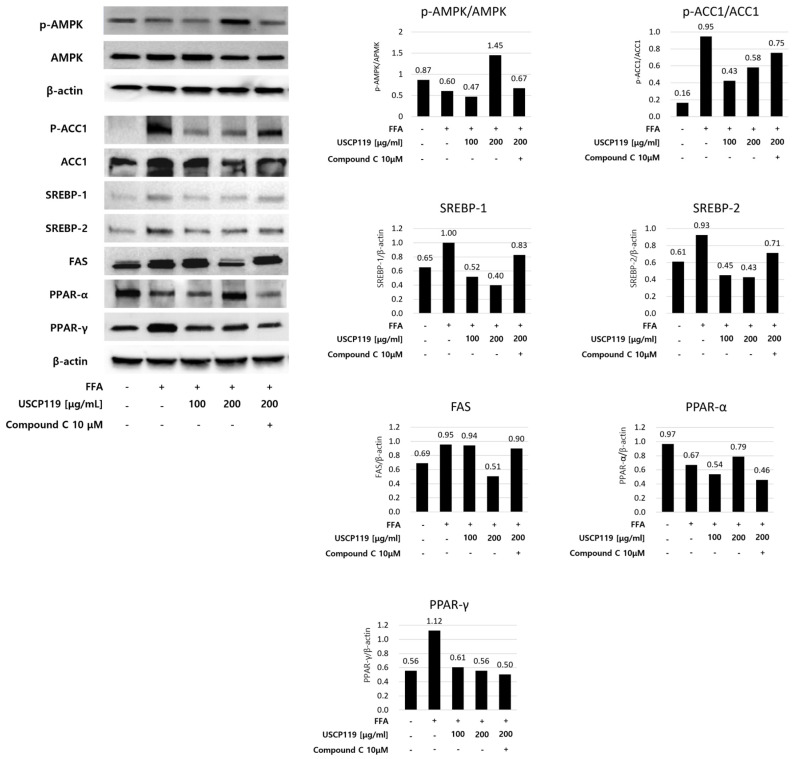
Effects of USCP119 on adipogenic protein expression in HepG2 cells. Data are expressed as mean ± SD of three independent experiments (*n* = 3).

To elucidate the mechanism underlying USCP119-mediated alterations in fatty acid metabolism in the liver, the phosphorylation of AMPK—a key signaling molecule involved in lipogenesis and lipolysis—as well as the expression of key proteins, including SREBP-1c, PPAR-γ, PPAR-α, FAS, and acetyl-CoA carboxylase (ACC) in the liver tissue were analyzed using Western blot analysis (Figure 8). Additionally, the impact of AMPK activation on the expression of PPAR-γ, PPAR-α, SREBP-1c, FAS, and ACC proteins was investigated by treating HepG2 cells with Compound C (also known as dorsomorphin), an AMPK inhibitor [26], either alone or in combination with USCP119. The results demonstrated that USCP119 promoted AMPK phosphorylation in HepG2 cells in a concentration-dependent manner. Furthermore, treatment with Compound C confirmed that USCP119 modulates AMPK signaling. In addition, proteins such as *p*-ACC1, SREBP-1, SREBP-2, and FAS, which were elevated after FFA treatment, were reduced post-USCP119 treatment, indicating an increase in PPAR-α expression. PPAR-α plays a crucial role in fat metabolism (fatty acid *β*-oxidation) by regulating the expression of proteins involved in the transport and *β*-oxidation of FFAs in liver tissue [27]. These proteins were down-regulated upon treatment with Compound C and USCP119, confirming that the observed changes in the expression of *p*-ACC1, SREBP-1, SREBP-2, FAS, and PPAR-α are attributed to AMPK signaling.

### 3.6. Effect of USCP119 on AMPK Signaling Pathways in the HFD Hamster Model

**Figure 9:**
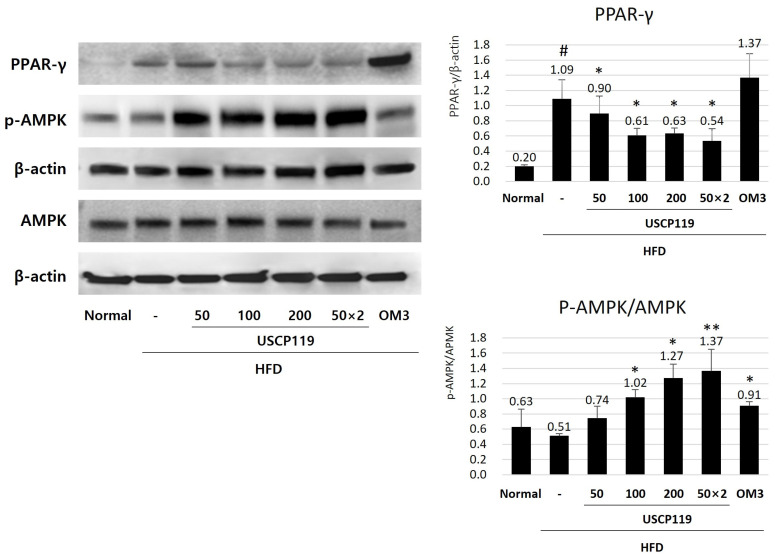
Effect of USCP119 on AMPK signaling pathways in the HFD hamster model. Data are expressed of three independent experiments (*n* = 3). ^#^ *p* < 0.05 compared with the ND group (G1); * *p* < 0.05 and ** *p* < 0.01 compared with the HFD group (G2).

The Western blot analysis (Figure 9) revealed that USCP119 treatment significantly upregulates phosphorylated AMPK levels in both normal and HFD hamster models, indicating potential activation of the AMPK signaling pathway. Additionally, the observed downregulation of PPAR-γ, a key transcription factor involved in adipogenesis and lipid metabolism [28], suggests that USCP119 may exert anti-hyperlipidemic effects. 

In comparison, the OM3-treated group showed greater activation of *p*-AMPK compared to the USCP119-treated groups. However, total AMPK levels remained consistent between the OM3 and USCP119 treatments, suggesting that differences in *p*-AMPK levels are due to changes in phosphorylation rather than total protein levels. Moreover, OM3 produced a more intense PPAR-γ band than the USCP119-treated group, indicating less effective downregulation of PPAR-γ, which may influence its efficacy in managing lipid levels. Overall, these findings imply that USCP119 is a promising therapeutic candidate for hypolipidemic effects, potentially offering benefits such as improved insulin sensitivity, reduced fat accumulation, and enhanced energy expenditure.

## 4. Discussion

Natural products have increasingly garnered attention for their potential to manage hyperlipidemia and related metabolic disorders [29,30]. Numerous natural compounds have been shown to influence BW, lipid profiles, hepatic lipid accumulation, and cellular lipid metabolism through various mechanisms, often by modulating key signaling pathways, such as AMPK [31,32,33]. This growing interest is driven by the quest for effective and safer alternatives to synthetic drugs for treating metabolic conditions.

Our previous study highlighted the therapeutic potential of a *S. miltiorrhiza* and *P. lactiflora* mixed extract for cardiovascular diseases, demonstrating its effectiveness in reducing vascular aging through both in vitro and in vivo experiments [34]. Specifically, this extract reduces vascular aging by inhibiting abnormal cell proliferation, collagen overproduction, and the overexpression of metalloproteinases, such as matrix metalloproteinase-2 (MMP-2) and MMP-9. Additionally, it restores superoxide dismutase activity, lowers intercellular and vascular cell adhesion molecule-1 levels, and prevents vascular wall aging. Building on these findings, the present study demonstrates the potential of *S. miltiorrhiza* and *P. lactiflora* as effective interventions for managing hyperlipidemia and metabolic diseases.

The data reveal that hamsters fed an HFD for 8 weeks exhibited a substantial increase in BW compared to those on a normal diet, confirming the weight-gain effects of high-fat intake. Notably, the addition of USCP119 to the HFD regimen resulted in varying degrees of weight reduction. The group receiving USCP119 at 50 mg/kg BID (G5) demonstrated the most pronounced effect, with significantly lower weight gain compared to other groups. This suggests that USCP119 can mitigate the weight-gain effects of HFD, highlighting its potential as a dietary supplement for weight management. Interestingly, while the 100 mg/kg dose of USCP119 resulted in a lower percentage of weight gain compared to the 50 mg/kg dose, it did not surpass the 50 mg/kg BID group (G5) in effectiveness. This differential response underscores the complexity of dosage optimization and suggests that the frequency of administration plays a crucial role in maximizing therapeutic benefits.

Research indicates that reducing LDL, TG, and TC levels while increasing HDL levels is directly linked to anti-hyperlipidemic effects and improved cardiovascular health [35,36,37]. USCP119 treatment led to significant reductions in serum TG and TC levels in the HFD hamster model. The notable decrease in TG levels, particularly at higher doses of USCP119, supports the hypothesis that USCP119 has a lipid-lowering effect. This is further corroborated by the observed reduction in LDL-C levels in the USCP119-treated groups. However, no significant differences in HDL-C levels were noted between the USCP119-treated and control groups. These findings align with observations from previous studies [35,36], suggesting that USCP119 may positively influence lipid metabolism and potentially reduce cardiovascular risk factors associated with obesity.

USCP119 treatment resulted in a reduction in liver weight and hepatic TG levels, particularly at the 50 mg/kg BID dose, indicating its effectiveness in counteracting hepatic fat accumulation associated with HFD. Oil Red O staining further confirmed significant lipid accumulation in the liver of HFD-fed hamsters, which was markedly reduced by USCP119 treatment. These observations underscore the protective effects of USCP119 on liver health, likely attributable to its ability to modulate lipid metabolism. Additionally, USCP119 treatment resulted in reduced epididymal fat mass and adipocyte diameter, with the most pronounced effects observed in the 50 mg/kg BID group (G5). This reduction in adipocyte hypertrophy is indicative of USCP119’s potential to prevent or mitigate obesity-related changes in fat distribution and accumulation.

**Figure 10:**
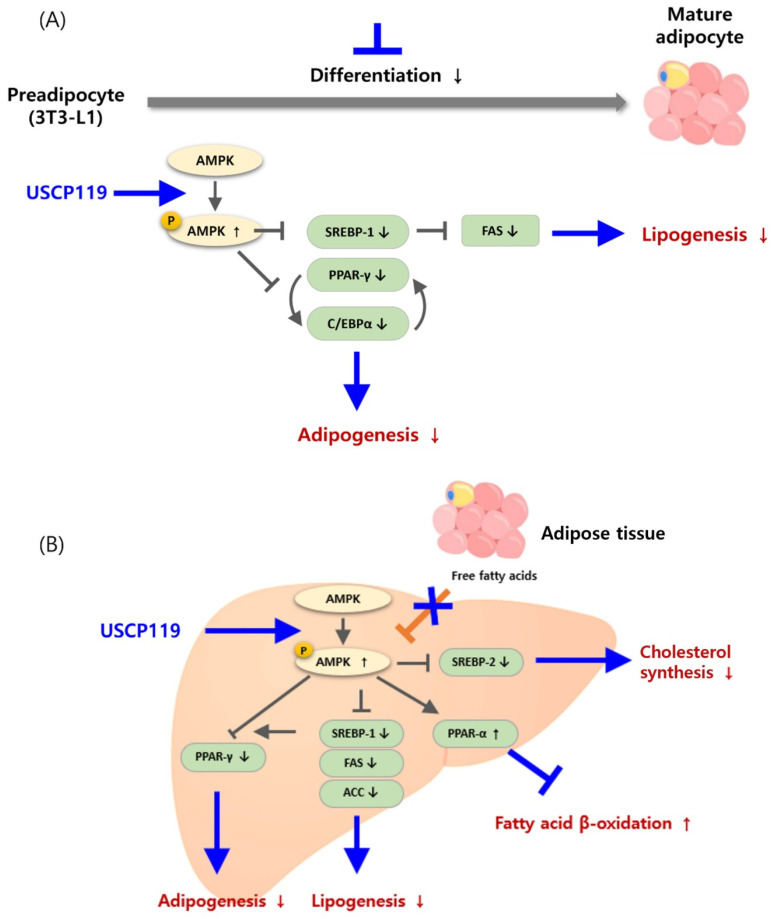
(**A**) Mechanism of USCP119-mediated regulation of adipocyte differentiation in 3T3-L1 cells: USCP119 inhibits adipogenesis by increasing the expression of activated AMPK and preventing the differentiation of adipocyte precursor cells into mature adipocytes. It does so by significantly decreasing the expression of key factors involved in the adipogenesis process, including PPARγ, C/EBPα, SREBP-1, and FAS, thereby reducing the accumulation of triglycerides in adipocytes. (**B**) Mechanism of USCP119-mediated regulation of triglycerides and fatty acids in HepG2 cells: treatment with USCP119 in adipocyte-induced HepG2 cells and liver-derived cells regulates the production of key factors and enzymes involved in fat metabolism, including SREBP-1, SREBP-2, PPARγ, C/EBPα, ACC, and FAS. USCP119 treatment also decreases the expression of *p*-AMPK and PPAR-α proteins. To investigate how AMPK activation affects the expression of PPAR-γ, PPAR-α, SREBP-1c, FAS, and ACC, HepG2 cells were treated with AMPK-selective inhibitors alone or in combination with USCP119. This experimental approach was used to assess the regulation of downstream protein expression by AMPK.

In cellular models, USCP119 demonstrated non-toxicity up to 400 µg/mL, establishing it as a safe candidate for further investigation. The observed reduction in lipid accumulation and TG content in both 3T3-L1 pre-adipocytes and HepG2 cells highlights USCP119’s effectiveness in inhibiting lipid accumulation and promoting lipid metabolism at the cellular level. The inhibition of adipogenic markers (PPAR-γ, C/EBPα, SREBP-1, and FAS) and the activation of AMPK signaling in 3T3-L1 cells further support USCP119’s role in preventing adipogenesis and enhancing lipolysis (Figure 10A). These results align with those observed in HepG2 cells, where USCP119’s impact on AMPK phosphorylation and regulation of key fatty acid metabolism proteins was observed (Figure 10B). 

The upregulation of phosphorylated AMPK levels in both normal and HFD-fed hamster models treated with USCP119 confirms the activation of the AMPK signaling pathway. This activation is associated with improved insulin sensitivity, reduced fat accumulation, and enhanced energy expenditure. Additionally, the downregulation of PPAR-γ in response to USCP119 treatment indicates its potential to counteract the effects of an HFD by modulating adipogenic pathways.

Despite the promising findings, this study has several limitations. First, the relatively small sample size and the use of an animal model may limit the generalizability of the results to human populations. Further studies using larger sample sizes and human clinical trials are necessary to validate the therapeutic potential of USCP119. Additionally, there were some challenges with the specificity of certain antibodies used in the Western blot analysis, particularly for FASN and ACC. The FASN antibody exhibited some non-specific binding, and ACC showed smeared bands, possibly due to post-translational modifications or incomplete resolution of the protein during electrophoresis. These limitations may have affected the precision of the results related to these specific proteins. Thus, future studies should optimize antibody conditions for better accuracy.

## 5. Conclusions

The results from both animal studies and cellular assays underscore the potential of USCP119 as a promising therapeutic agent for managing hyperlipidemia and metabolic diseases. USCP119’s efficacy in modulating AMPK signaling and influencing key lipid-regulating proteins highlights its role in altering lipid metabolism and reducing fat accumulation. Overall, this study demonstrates that USCP119 reduces BW, improves lipid profiles, and modulates critical signaling pathways involved in metabolic dysfunction, positioning USCP119 as a potentially valuable strategy for addressing hyperlipidemia.

## Figures and Tables

**Table 1:** Body weight changes in hamsters fed a normal diet (ND) or high-fat diet (HFD) with or without USCP119 or OM3 supplementation over 8 weeks.

Group	Time after Administration (Weeks)			
	Body Weight (g)			% of BW Change (8 w/0 w × 100%)
	0 w	4 w	8 w	
G1—Normal diet	108.8 ± 6.7	120.4 ± 8.4	126.9 ± 8.8	18.1 ± 10.5
G2—HFD	108.4 ± 5.6	127.9 ± 11.5	138.2 ± 6.1	29.8 ± 10.2
G3—HFD + USCP119_50	105.1 ± 6.1	121.7 ± 13.4	131.1 ± 13.4	29.0 ± 12.3
G4—HFD + USCP119_100	106.7 ± 5.9	121.9 ± 6.2	132.0 ± 7.7	25.4 ± 7.7
G5—HFD + USCP119_50 × 2	103.7 ± 8.6	113.1 ± 6.9 *	119.2 ± 7.9 *	15.5 ± 9.6 *
G6—HFD + OM3	106.3 ± 6.6	121.1 ± 11.3	127.7 ± 13.1	21.3 ± 16.3

Data represent mean ± SD (*n* = 7 for each group). * *p* < 0.05 compared with the HFD group.

**Table 2:** Effects of USCP119 on serum lipid profiles in the HFD hamster model, including the levels of serum triglycerides (TG), serum total cholesterol (TC), serum high-density lipoprotein cholesterol (HDL-C), and serum low-density lipoprotein cholesterol (LDL-C).

Group	Serum TG Level (mmol/L)	Serum TC Level (mmol/L)	Serum HDL-C Level (mmol/L)	Serum LDL-C Level (mmol/L)
G1	120.0 ± 14.63	99.86 ± 8.88	68.14 ± 6.69	18.43 ± 1.27
G2	287.29 ± 24.64 ^###^	280.29 ± 30.46 ^###^	131.00 ± 14.40 ^###^	33.29 ± 13.47 ^#^
G3	248.43 ± 36.02 *	265.00 ± 22.46	121.71 ± 11.91	30.00 ± 5.80
G4	216.43 ± 19.90 ***	227.57 ± 36.41 *	109.86 ± 18.17	20.57 ± 5.50 *
G5	184.29 ± 17.98 ***	240.14 ± 22.59 *	117.57 ± 9.88	25.14 ± 8.03
G6	244.14 ± 34.57 *	275.00 ± 33.26	117.57 ± 20.70	43.86 ± 8.61

Data are expressed as mean ± SD; ^#^ *p* < 0.05 and ^###^ *p* < 0.001 compared with the ND group (G1); * *p* < 0.05 and *** *p* < 0.001 compared with the HFD group (G2).

**Table 3:** Effects of USCP119 on hepatic lipid and epididymal fat levels in the HFD hamster model.

Group	Liver Weight (g)	Hepatic TG Level (µg/mg)	Oil Red O-Positive Intensity (Arbitrary Unit)	Epididymal Fat Weight (g)	eWAT Lipid Diameter (µm)
G1	3.30 ± 0.14	28.54 ± 4.69	50.38 ± 8.99	1.70 ± 0.38	48.06 ± 4.92
G2	8.37 ± 0.96 ^##^	45.85 ± 8.26 ^##^	223.77 ± 8.34 ^###^	2.56 ± 0.64 ^#^	61.11 ± 7.26 ^#^
G3	8.37 ± 0.94	34.56 ± 4.33 *	210.69 ± 7.88 *	2.50 ± 0.86	51.87 ± 5.37
G4	8.16 ± 0.50	29.55 ± 5.49 **	202.06 ± 20.41 *	2.40 ± 0.69	51.20 ± 4.44
G5	7.27 ± 0.60 *	28.01 ± 6.11 **	205.81 ± 15.13 *	1.85 ± 0.42 *	50.97 ± 3.97 *
G6	8.46 ± 1.04	37.97 ± 3.17	214.06 ± 3.53 *	2.41 ± 0.64	59.78 ± 5.05

Data are expressed as mean ± SD; ^#^ *p* < 0.05, ^##^ *p* < 0.01, and ^###^ *p* < 0.001 compared with the ND group (G1); * *p* < 0.05 and ** *p* < 0.01 and compared with the HFD group (G2).

## Data Availability

Data that supports this study are available in the Article.

## Supplementary Materials

The following supporting information can be downloaded at: https://www.mdpi.com/article/10.3390/nu16183189/s1, Detailed protocols for liver TG quantification, cell culture, cell viability and proliferation assays, Western blot analysis, and AMPK phosphorylation inhibition are provided in the Supplemental Material.

## References

[1] Tsuru H., Osaka M., Hiraoka Y., Yoshida M. (2020). HFD-induced hepatic lipid accumulation and inflammation are decreased in Factor D deficient mouse. Sci. Rep..

[2] Dong Y., Guo Y., Li Q., Zhao Y., Cao J. (2024). Soluble dietary fiber from *Dendrocalamus brandisii* (Munro) Kurz shoot improves liver injury by regulating gut microbial disorder in mice. Food Chem. X.

[3] Yin Y., Li Z., Gao L., Li Y., Zhao J., Zhang W. (2015). AMPK-dependent modulation of hepatic lipid metabolism by nesfatin-1. Mol. Cell. Endocrinol..

[4] Semova I., Biddinger S.B. (2021). Triglycerides in nonalcoholic fatty liver disease: Guilty until proven innocent. Trends Pharmacol. Sci..

[5] Rodríguez-Lara A., Rueda-Robles A., Sáez-Lara M.J., Plaza-Diaz J., Álvarez-Mercado A.I. (2023). From non-alcoholic fatty liver disease to liver cancer: Microbiota and inflammation as key players. Pathogens.

[6] Park W.Y., Yiannakou I., Petersen J.M., Hoffmann U., Ma J., Long M.T. (2022). Sugar-sweetened beverage, diet soda, and nonalcoholic fatty liver disease over 6 years: The framingham heart study. Clin. Gastroenterol. Hepatol..

[7] Naomi N.D., Ngo J., Brouwer-Brolsma E.M., Buso M.E.C., Soedamah-Muthu S.S., Pérez-Rodrigo C., Harrold J.A., Halford J.C.G., Raben A., Geleijnse J.M. (2023). Sugar-sweetened beverages, low/no-calorie beverages, fruit juice and non-alcoholic fatty liver disease defined by fatty liver index: The SWEET project. Nutr. Diabetes.

[8] Chen Y., Yu C.-Y., Deng W.-M. (2019). The role of pro-inflammatory cytokines in lipid metabolism of metabolic diseases. Int. Rev. Immunol..

[9] Deprince A., Haas J.T., Staels B. (2020). Dysregulated lipid metabolism links NAFLD to cardiovascular disease. Mol. Metab..

[10] Lee M.-H., Park S., Xu Y., Kim J.-E., Han H., Lee J.-H., Paik J.K., Lee H.-J. (2022). Ethanol extract of *Pinus koraiensis* leaves mitigates high fructose-induced hepatic triglyceride accumulation and hypertriglyceridemia. Appl. Sci..

[11] Zhang G.H., Lu J.X., Chen Y., Guo P.H., Qiao Z.L., Feng R.F., Chen S.E., Bai J.L., Huo S.D., Ma Z.R. (2015). ChREBP and LXRα mediate synergistically lipogenesis induced by glucose in porcine adipocytes. Gene.

[12] Lee M.-S., Cho S.-M., Lee M.-h., Lee E.-O., Kim S.-H., Lee H.-J. (2016). Ethanol extract of *Pinus koraiensis* leaves containing lambertianic acid exerts anti-obesity and hypolipidemic effects by activating adenosine monophosphate-activated protein kinase (AMPK). BMC Complement. Altern. Med..

[13] Rodríguez C., Muñoz M., Contreras C., Prieto D. (2021). AMPK, metabolism, and vascular function. FEBS J..

[14] Huang Y., Xie H., Pan P., Qu Q., Xia Q., Gao X., Zhang S., Jiang Q. (2021). Heat stress promotes lipid accumulation by inhibiting the AMPK-PGC-1α signaling pathway in 3T3-L1 preadipocytes. Cell Stress Chaperones.

[15] Smith B.K., Marcinko K., Desjardins E.M., Lally J.S., Ford R.J., Steinberg G.R. (2016). Treatment of nonalcoholic fatty liver disease: Role of AMPK. Am. J. Physiol.-Endocrinol. Metab..

[16] Jung I., Kim H., Moon S., Lee H., Kim B. (2020). Overview of *Salvia miltiorrhiza* as a potential therapeutic agent for various diseases: An update on efficacy and mechanisms of action. Antioxidants.

[17] Parker S., May B., Zhang C., Zhang A.L., Lu C., Xue C.C. (2016). A pharmacological review of bioactive constituents of *Paeonia lactiflora* Pallas and *Paeonia veitchii* Lynch. Phytother. Res..

[18] Wang X., Wang Y., Jiang M., Zhu Y., Hu L., Fan G., Wang Y., Li X., Gao X. (2011). Differential cardioprotective effects of salvianolic acid and tanshinone on acute myocardial infarction are mediated by unique signaling pathways. J. Ethnopharmacol..

[19] Pan C., Lou L., Huo Y., Singh G., Chen M., Zhang D., Wu A., Zhao M., Wang S., Li J. (2011). Salvianolic acid B and tanshinone IIA attenuate myocardial ischemia injury in mice by NO production through multiple pathways. Ther. Adv. Cardiovasc. Dis..

[20] Nam K.N., Woo B.-C., Moon S.-K., Park S.-U., Park J.-Y., Hwang J.-W., Bae H.-S., Ko C.-N., Lee E.H. (2013). Paeonol attenuates inflammation-mediated neurotoxicity and microglial activation. Neural Regen. Res..

[21] Wang D., Liu L., Li S., Wang C. (2018). Effects of paeoniflorin on neurobehavior, oxidative stress, brain insulin signaling, and synaptic alterations in intracerebroventricular streptozotocin-induced cognitive impairment in mice. Physiol. Behav..

[22] Reeves P.G. (1997). Components of the AIN-93 diets as improvements in the AIN-76A diet. J. Nutr..

[23] Trang N.M., Kim E.-N., Lee H.-S., Jeong G.-S. (2022). Effect on osteoclast differentiation and ER stress downregulation by amygdalin and RANKL binding interaction. Biomolecules.

[24] Trang N.M., Kim E.-N., Pham T.H., Jeong G.-S. (2023). Citropten ameliorates osteoclastogenesis related to MAPK and PLCγ/Ca^2+^ signaling pathways through the regulation of amyloid beta. J. Agric. Food Chem..

[25] Ali K.M., Wonnerth A., Huber K., Wojta J. (2012). Cardiovascular disease risk reduction by raising HDL cholesterol—Current therapies and future opportunities. Br. J. Pharmacol..

[26] Liu X., Chhipa R.R., Nakano I., Dasgupta B. (2014). The AMPK inhibitor Compound C is a potent AMPK-independent antiglioma agent. Mol. Cancer Ther..

[27] Pawlak M., Lefebvre P., Staels B. (2015). Molecular mechanism of PPARα action and its impact on lipid metabolism, inflammation and fibrosis in non-alcoholic fatty liver disease. J. Hepatol..

[28] Corrales P., Vidal-Puig A., Medina-Gómez G. (2018). PPARs and metabolic disorders associated with challenged adipose tissue plasticity. Int. J. Mol. Sci..

[29] Shaik Mohamed Sayed U.F., Moshawih S., Goh H.P., Kifli N., Gupta G., Singh S.K., Chellappan D.K., Dua K., Hermansyah A., Ser H.L. (2023). Natural products as novel anti-obesity agents: Insights into mechanisms of action and potential for therapeutic management. Front. Pharmacol..

[30] Majeed M., Majeed S., Nagabhushanam K., Gnanamani M., Mundkur L. (2021). Lesser investigated natural ingredients for the management of obesity. Nutrients.

[31] Li H., Rafie A.R., Hamama A., Siddiqui R.A. (2023). Immature ginger reduces triglyceride accumulation by downregulating Acyl CoA carboxylase and phosphoenolpyruvate carboxykinase-1 genes in 3T3-L1 adipocytes. Food Nutr. Res..

[32] Li J., Liu M., Yu H., Wang W., Han L., Chen Q., Ruan J., Wen S., Zhang Y., Wang T. (2018). Mangiferin improves hepatic lipid metabolism mainly through its metabolite-norathyriol by modulating SIRT-1/AMPK/SREBP-1c signaling. Front. Pharmacol..

[33] Bort A., Sánchez B.G., Mateos-Gómez P.A., Díaz-Laviada I., Rodríguez-Henche N. (2019). Capsaicin targets lipogenesis in HepG2 cells through AMPK activation, AKT inhibition and PPARs regulation. Int. J. Mol. Sci..

[34] Son J., Cha M.-R., Song S., Oh B., Bang S., Cha J., Lim S.D., Yang S.Y. (2024). Efficacy of a mixed extract of *Salvia miltiorrhiza* and *Paeonia lactiflora* in inhibiting the aging of vascular wall through in vitro and in vivo experiments. Biosci. Biotechnol. Biochem..

[35] Kucukkurt I., Akkol E.K., Karabag F., Ince S., Süntar I., Eryavuz A., Sözbilir N.B. (2016). Determination of the regulatory properties of *Yucca schidigera* extracts on the biochemical parameters and plasma hormone levels associated with obesity. Rev. Bras. Farmacogn..

[36] Shrestha R., Gurung P., Lim J., Thapa Magar T.B., Kim C.-W., Lee H.Y., Kim Y.-W. (2023). Anti-obesity effect of Chlorin e6-mediated photodynamic therapy on mice with high-fat-diet-induced obesity. Pharmaceuticals.

[37] Cherng J.-Y., Shih M.-F. (2005). Preventing dyslipidemia by *Chlorella pyrenoidosa* in rats and hamsters after chronic high fat diet treatment. Life Sci..

