# Supplementary material for: AMPK-Mediated Hypolipidemic Effects of a Salvia miltiorrhiza and Paeonia lactiflora Mixed Extract on High-Fat Diet-Induced Liver Triglyceride Accumulation: An In Vivo and In Vitro Study
Source: Nutrients. 2024 Sep 20;16(18):3189. doi: 10.3390/nu16183189 (PMC11434907; doi:10.3390/nu16183189)
Supplement: Supplementary file 1 [file nutrients-16-03189-s001.zip › nutrients-3195651-supplementary.pdf]

SUPPLEMENTARY MATERIAL

AMPK-Mediated Hypolipidemic Effects of a *Salvia miltiorrhiza* and *Paeonia lactiflora* Mixed Extract on High-Fat Diet-Induced Liver Triglyceride Accumulation: An In Vivo and In Vitro Study

Juah Son [1,†], Nguyen Viet Phong [2,†], Mi-Ran Cha [1], Byulnim Oh [1], Sukjin Song [1,*], and Seo Young Yang [2,*]

[1] USCAREPHARM Co., Ltd., Suwon 16690, Republic of Korea; juah@uscarepharm.com (J.S.), mrcha@uscarepharm.com (M.-R.C.), aster@uscarepharm.com (B.O.)

[2] Department of Biology Education, Teachers College and Institute for Phylogenomics and Evolution, Kyungpook National University, Daegu 41566, Republic of Korea; ngvietphong@gmail.com

* Correspondence: uscare@uscarepharm.com (S.S.), syy@knu.ac.kr (S.Y.Y.); Tel.: +82-31-8081-1136 (S.S.), +82-53-950-5910 (S.Y.Y.)

† These authors contributed equally to this work.

1. Measurement of liver TG levels

Liver samples were collected after perfusion with 0.1 M phosphate-buffered saline (PBS), immediately frozen on dry ice, and stored at −80 °C until analysis. Liver TG concentration was measured using a TG quantification kit (Sigma-Aldrich, St. Louis, MO, USA), following the manufacturer's instructions. Briefly, liver tissue samples (100 mg) were homogenized in 1 mL of 5% Nonidet-P40 substitute (Sigma-Aldrich). Subsequently, the samples were transferred to a 96-well plate and heated to 80–100 °C for 2–5 min and then cooled to room temperature (RT; 20–25 °C); this heating and cooling step was repeated once. Then, lipase was added to convert the TGs to glycerol and fatty acids, followed by the addition of the master reaction mix to each well to complete the reaction. Finally, colorimetric detection was performed by measuring the absorbance at 570 nm.

2. Cell culture

3T3-L1 cells (murine preadipocytes of fibroblast cell line) and HepG2 cells (human hepatoma cell line) were purchased from the American Type Culture Collection (Manassas, VA, USA). The 3T3-L1 cells were cultured in DMEM supplemented with 10% bovine serum (BS) and 1% penicillin-streptomycin (P/S). HepG2 cells were cultured in DMEM supplemented with 10% fetal bovine serum (FBS), 2% P/S, and 2% HEPES. Both cell lines were maintained at 37 °C in a 5% CO_2 atmosphere. For both cell lines, media renewal was performed every 2–3 days, and sub-culturing was performed until the cells reached 80% confluence.

2.1. Differentiation of 3T3-L1 cells

Pre-adipocyte 3T3-L1 cells (1×10^5 cells/well) were seeded in 6-well plates and cultured in BS media until they reached 100% confluence. Subsequently, the medium was replaced with a differentiation medium containing 10% FBS, 1% P/S, 1 μM dexamethasone, 0.5 mM 3-isobutyl-1-methylxanthine (IBMX), and 10 μg/mL insulin to induce differentiation. The sample was maintained in an incubator set at 37 °C with 5% CO_2. Two days after inducing differentiation, the medium was changed to DMEM supplemented with 10% FBS, 1% P/S, and 10 μg/mL insulin, and USCP119 treatment was performed three times (once every two days). After 5–7 days of inducing differentiation, approximately 90% of the pre-adipocytes had transformed into round-shaped mature fat cells.

2.2. Free fatty acids-induced steatosis in HepG2 cells

A free fatty acid (FFA) solution was prepared by dissolving oleic acid and palmitic acid (2:1 ratio) in isopropyl alcohol. This FFA solution (5 mM) was then added to serum-free DMEM containing 5% fatty acid-free bovine serum albumin (BSA). HepG2 cells were seeded (1×10^5 cells/mL) in 12-well plates containing the culture media. Once the cells reached 80–90% confluence, they were treated with serum-free media containing the FFA solution (0.5 mM) and USCP119 for 24 h to induce intracellular lipid accumulation.

2.3. Analysis of cellular lipid accumulation via Oil Red O staining

Oil Red O staining was performed to examine the morphology and total lipid accumulation in the HepG2 and 3T3-L1 cells. The Oil Red O stock solution was prepared by dissolving Oil Red O in 0.5% isopropanol and then diluting it with isopropanol and water (6:4). Cells were first washed twice with PBS and then fixed with 4% paraformaldehyde at RT (HepG2 cells were fixed for 30 min, while 3T3-L1 cells were fixed for 60 min). Subsequently, the cells were rinsed with PBS and stained with Oil Red O for 30 min at RT. After staining, the cells were washed again to remove any excess dye. Images of the stained cells were then captured using an inverted microscope (Nikon, Tokyo, Japan). Subsequently, the dyed lipids were quantified by dissolving the cells in isopropanol. The resulting solution (200 μL) was transferred to a 96-well culture plate, and the absorbance was measured at 510 nm to assess accumulation.

2.4. Cellular TG content

Cellular TG content was determined using a TG quantification kit (MAK266, Sigma-Aldrich), following the manufacturer's instructions. Briefly, 3T3L1 or HepG2 cells were cultured and induced for adipogenesis or steatosis as previously described. The experiment was performed in triplicate for each condition. After treatment, the cells were detached from the culture flasks, counted, and assessed for viability.

3. Cell viability and proliferation assay

Cell viability and proliferation were assessed using the water-soluble tetrazolium (WST)-8 assay (Biomax, Nowon, Seoul, Korea). The WST-8 assay, which does not involve dimethyl sulfoxide (DMSO), avoids potential cell damage associated with DMSO and offers high sensitivity for accurate measurement of cell viability. Briefly, cells were seeded in 96-well plates (1×10^5 cells/well) and incubated for 24 h. After serum starvation for 24 h, the cells were treated with USCP119 at concentrations of 50, 100, 200, and 400 μg/mL and incubated for an additional 72 h. Subsequently, the WST-8 reagent was added, and the plates were incubated for 2 h. Finally, the absorbance at 450 nm was measured using a Multiskan FC microplate reader (Thermo Fisher Scientific, Waltham, MA, USA). Cell proliferation is presented as a relative change compared to control.

4. Western blot analysis

Total protein was extracted using RIPA protein extraction reagent supplemented with a protease inhibitor cocktail (P8340, Sigma-Aldrich). Protein concentration was measured using a BCA protein assay kit (Biomax, Seoul, Korea). Equal amounts of protein extract were loaded onto 4–15% mini-Protean TGX stain-free gels (Bio-Rad, Hercules, CA, USA) for separation and then transferred to Immun-Blo PVDF membranes (Bio-Rad). The membranes were then incubated with 5% non-fat dry milk (M7409, Sigma-Aldrich) for 1 h to block non-specific binding. Then, primary antibodies against PPAR-α, CCAAT/enhancer-binding protein alpha (C/EBPα), SREBP-1c, SREBP-2, FAS, PPAR-γ, acetyl CoA carboxylase (ACC), p-ACC, AMPK, and p-AMPK were applied, and the membranes were incubated overnight at 4 °C. After washing, the membranes were incubated with anti-rabbit or anti-mouse IgG antibodies conjugated with horseradish peroxidase for 2 h at RT. Immunostaining was visualized using WestGlow PICO PLUS ECL chemiluminescent substrate (Biomax). PPAR-α (600-401-421) was purchased from Rockland Immunochemicals Inc. (Limerick, PA, USA). C/EBPα (#2295), PPAR-γ (#2492), ACC (#3662), p-ACC (#3661), AMPK (#2532), and p-AMPK (#2535) were purchased from Cell Signaling Technology (Beverly, MA, USA). SREBP-1c (sc-13551), SREBP-2 (sc-13552), FAS (sc-21730) were purchased from Santa Cruz Biotechnology (Santa Cruz, CA, USA).

5. Inhibition of AMPK phosphorylation

Following 12 h of starvation, the fatty HepG2 cells were treated with 10 μM compound C (dorsomorphin) or DMSO for 1 h. Subsequently, these cells were treated with USCP119 or DMSO. The expressions of phospho-AMPK, AMPK, phospho-ACC, ACC, FAS, SREBP-1, SREBP-2, PPAR-α, PPAR-γ, and β-actin were analyzed using the Western blot procedure described in Section 4 (Supplemental Material).
